# Meditation Effects on the Control of Involuntary Contingent Reorienting Revealed With Electroencephalographic and Behavioral Evidence

**DOI:** 10.3389/fnint.2018.00017

**Published:** 2018-05-15

**Authors:** Shao-Yang Tsai, Satish Jaiswal, Chi-Fu Chang, Wei-Kuang Liang, Neil G. Muggleton, Chi-Hung Juan

**Affiliations:** ^1^Institute of Cognitive Neuroscience, National Central University, Taoyuan, Taiwan; ^2^Brain Research Center, National Central University, Taoyuan, Taiwan; ^3^Institute of Cognitive Neuroscience, University College London, London, United Kingdom; ^4^Department of Psychology, Goldsmiths, University of London, London, United Kingdom

**Keywords:** meditation, contingent reorienting, attentional control, attentional suppression, event related modes

## Abstract

Prior studies have reported that meditation may improve cognitive functions and those related to attention in particular. Here, the dynamic process of attentional control, which allows subjects to focus attention on their current interests, was investigated. Concentrative meditation aims to cultivate the abilities of continuous focus and redirecting attention from distractions to the object of focus during meditation. However, it remains unclear how meditation may influence attentional reorientation, which involves interaction between both top-down and bottom-up processes. We aimed to investigate the modulating effect of meditation on the mechanisms of contingent reorienting by employing a rapid serial visual presentation (RSVP) task in conjunction with electrophysiological recording. We recruited 26 meditators who had an average of 2.9 years of meditation experience and a control group comprising 26 individuals without any prior experience of meditation. All subjects performed a 30-min meditation and a rest condition with data collected pre- and post-intervention, with each intervention given on different days. The state effect of meditation improved overall accuracy for all subjects irrespective of their group. A group difference was observed across interventions, showing that meditators were more accurate and more efficient at attentional suppression, represented by a larger Pd (distractor positive) amplitude of event related modes (ERMs), for target-like distractors than the control group. The findings suggested that better attentional control with respect to distractors might be facilitated by acquiring experience of and skills related to meditation training.

## Background

In recent years research interest in meditation effects on brain function has increased exponentially (Hölzel et al., 2011; Tang et al., 2015). Meditation has been reported to have several beneficial effects on cognitive functions, presumably by altering one or more of the three core cognitive components: attentional networks, emotional control and self-awareness (Tang et al., 2015). Meditation aims to cultivate abilities to manipulate the orientation of attention, to monitor, detect and disengage from distractors, and to reorient attention toward a chosen object (Dahl et al., 2015). However, there has been relatively little research on meditation investigating the neural mechanisms involved in attentional control which underlying interference by salient but task-irrelevant distractors which induce contingent reorienting, a process that is a good candidate for any alteration by meditation.

Contingent reorienting can be defined as attention being captured by a salient stimulus that shares a defined characteristic with the target (Folk et al., 1992, 2002; Corbetta et al., 2008; Theeuwes, 2010). This process is linked with the limit to human cognitive resources, and the fact that we cannot process all stimuli around us. Attentional control is thought help to select or process the information which is important at a particular time. According to Moore et al. (2012), meditation practice improves self-regulation of attention and may increase efficiency of allocation cognitive resources. The main question of this study is whether and, if so, how meditation modulates attentional control. According to Sawaki and Luck (2014), a mechanism to prevent attention from being captured by salient but task-irrelevant objects is necessary. As such, meditation effects may be potentially linked to two forms of control: (1) control over the ability to prevent a stimulus from capturing attention; and (2) control over the ability to disengage attention from stimuli that do capture attention. It is unclear whether meditation increases the efficiency of shifting attention back from a distractor to a target or whether it affects the disengagement of attention from such a distractor. Shifting attention back from distractors might be specifically related to enhancing attention to relevant items, suppressing attention to irrelevant items, or both.

Many previous studies have investigated meditation effects and some benefits on attention have been reported, with a primary focus on the attentional network system, including alerting, orienting and executive control (e.g., Tang et al., 2007; van den Hurk et al., 2010; Baijal et al., 2011; Jo et al., 2016). One possibility is that meditation training could have the effect of enhancing attentional stability (Lutz et al., 2009) with Lutz et al. (2009) suggesting meditation training may relate to better cognitive control, such as an increased ability to suppress task-unrelated thoughts or distractions. However, little is known about the mechanisms and training effects of meditation on control over involuntary capture of attention by distractors and it is not clear which specific aspect of attentional control meditation may affect. When an unexpected distractor does capture attention, it is necessary to withdraw attention from it and back to the primary (relevant) object (Geng, 2014). Sawaki and Luck (2013) looked at how people reoriented attention to a target after involuntary capture by a distractor and found attentional suppression is critical in disengaging from distractors. Here, we expected that, should meditation training alter performance, such a process would be a good candidate for the origin of such modulation. Hence, this study aimed to examine the effects of meditation on attentional control of contingent reorienting that emphasized the interaction between goal-directed and stimulus-driven attentional control (Corbetta et al., 2008; Chang et al., 2013, 2016; Tsai et al., 2017).

Prior research involving meditation has suggested that orienting might be critical because, as part of meditation training, meditators are encouraged to not only monitor the quality of attention but also reorient attention during mind wandering (Elliott et al., 2014). For example, and according to Malinowski (2013), orientation of awareness is an important skill in meditation training. Meditators are instructed to let go of distracting thoughts, presumably through attentional disengagement and involvement of the executive network, when the mind wanders, and shift the focus of attention back to the object (Malinowski, 2013). However, it is unknown whether meditation reduces distraction from the target, if it facilitates disengagement of attention from a distractor or affects refocusing on the target. This study therefore attempted to apply a suitable paradigm for studying the relationship between meditation and attentional reorientation with a focus on both contingent reorienting and attentional suppression of distractors.

The specific aims were to identify: (1) whether meditators perform differently on a task involving attentional reorienting than non-meditators; (2) whether state induction of meditation compared to rest resulted in better or different behavioral performance; and (3) any physiological changes or differences associated with meditation. The design of the rapid serial visual presentation (RSVP) task employed in this study allowed investigation of meditation effects on attentional suppression of salient but task-irrelevant distractors which typically induce contingent reorienting (i.e., a shift of attention from a central target stream to laterally located distractors, see “Materials and Methods” section and Figure 2) and disengagement from the current attentional focus (e.g., shifting attention from a lateral distractor back to the central target stream). Previous studies using the RSVP paradigm (Chang et al., 2016; Tsai et al., 2017) demonstrated significant N2pc and Pd components in event related potential recordings during contingent reorienting. The N2pc is an index of attentional deployment (Sawaki and Luck, 2014), and is defined as the negative component around 200 ms following a stimulus, with “pc” referring to its posterior-contralateral location (Luck and Hillyard, 1994a). This component is more negative in the contralateral scalp area than the ipsilateral scalp area relative to the location of an attended object in a visual search display. The Pd (distractor positivity) component is thought to reflect an attentional suppression process involved in preventing and terminating attention (Sawaki and Luck, 2014). This component has a wide temporal range (around 100–400 ms) and depends on the types of stimuli and task used (Sawaki et al., 2012). The N2pc and Pd components have the potential to reveal specific processes and the magnitude of differential attentional capture effects in either two different populations (meditators and controls) or two different state inductions (short interventions of meditation or rest).

**Figure 1:**
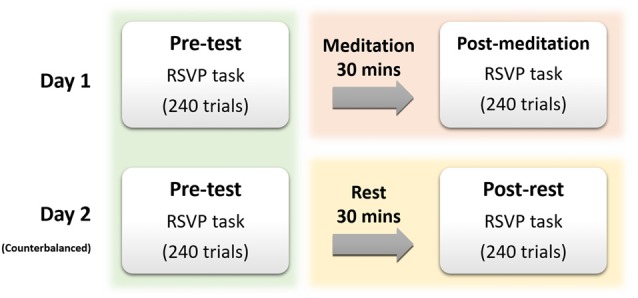
Experimental time-line. Two days of testing were used for each participant, with the order of meditation and rest balanced for groups and participants.

**Figure 2:**
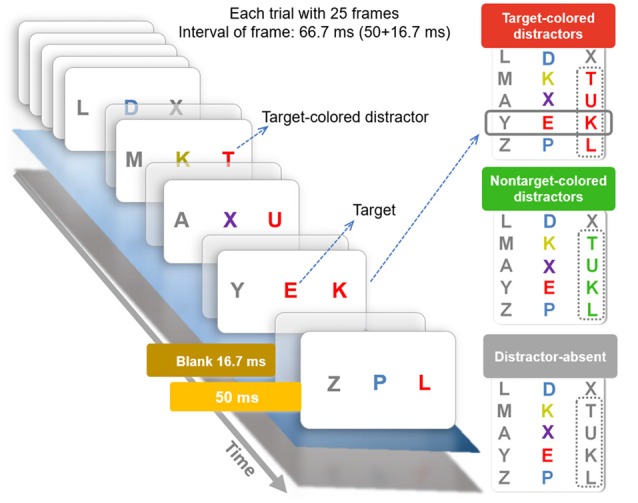
The rapid serial visual presentation (RSVP) task conditions and time-line. The left part of the figure shows the time-line of a typical trial. Each new letter was presented for 50 ms and followed by a 16.7 ms blank screen, such that letters appeared every 67 ms. The right part shows the three different trial type/conditions used in the task which were: (1) distractor-absent: the distractor stream was of just gray letters; (2) target-colored distractor: one peripheral stream contained red distractor letters which started two letters before the onset of the target in the central stream and continued until one letter after the target, making four letters in total; and (3) non-target-colored distractor: this was the same as the target-colored distractor condition except the peripheral distractor was green.

We hypothesized that meditation might be associated with both a state effect (i.e., an effect immediately following completion of meditation) and a cross-sectional effect (a difference between meditators and control participants) such that there would be differences in the efficiency of attentional control during distractor induced contingent reorienting. For the state effect, short meditation (compared to rest) might improve attentional performance. For the cross-sectional effect, due to the regular meditation training which presumably improves attentional control, meditators might have better ability to deal with interference from distractors than non-meditators. Thus, performance might be less interfered with by distractors in the meditator group compared to controls and there would be differences in the electrophysiological patterns consistent with this.

## Materials and Methods

An attentional capture task employing a RSVP paradigm (Chang et al., 2013, 2016) was used. This involved systematically manipulating three types of distractors to allow assessment of the attentional processing involved in contingent reorienting. The three independent variables in the experiment were: (1) group to which participants belonged (mediators or non-meditators); (2) intervention type employed (meditation or rest); and (3) distractor type in the RSVP task (distractor absent, non-target color (NTC) distractors, or target color (TC) distractors). Dependent variables were accuracy of RSVP task performance and EEG-related indices.

### Participants

All subjects were healthy college students with no history of psychiatric or neurological disorders and normal or corrected to normal vision, recruited from Taoyuan City, Taiwan (8 meditators from Chang Gung University, 2 meditators from Chung Yuan Christian University, 3 meditators from Kainan University, 1 meditator from National Taipei University of Technology (but living in Taoyuan) and remaining participants from National Central University). The meditation group consisted of 11 males and 15 females (mean age = 22.59 years, SD = 2.59, range: 19–30 years of age) and the control/non-meditator group was also 11 males and 15 females (mean age = 21.44 years, SD = 2.06, range: 18–25 years old). The meditators had training in Heart Chan meditation, one kind of concentrative Buddhist meditation, for at least 1 year with an average duration of practice of meditation of 2.92 years (SD = 1.62, range: 1–7 years). All the meditators took meditation classes regularly once a week from the Shakyamuni Buddhist Foundation in Taiwan. According to a survey of recent meditation practice of the meditators who participated in this study, there was an average of 4.12 practice times per week (SD = 1.75, range: 2–7 times) with an average duration of each practice of 32.69 min (SD = 11.07, range: 15–55 min). The matched controls had no prior experience of meditation, yoga, taichi or qigong. Trait and state personality characteristics were measured to assess individual variability within and across the groups that might be of relevance when analyzing the behavioral performance and electrophysiology measures. Before the experiment took place, participants received instruction regarding the experimental procedures and informed consent was obtained in accordance with the Declaration of Helsinki by means of a written consent form which also informed participants of their right to quit the experiment without reason at any time. All experimental procedures were approved by the Research Ethics Office of National Taiwan University, Taipei, Taiwan.

### Questionnaires

There were five online questionnaires that all participants had to complete on the first day before taking part in the experiment. The first was the anxiety state section of the State-Trait Anxiety Inventory (STAI-state; Spielberger et al., 1983), giving the “state” anxiety level on the first experimental day. The other questionnaires all measured relatively longitudinal traits. These were the State-Trait Anxiety Inventory (STAI-trait), the Mindful Attention Awareness Scale (MAAS; Brown and Ryan, 2003; Chang et al., 2011), the Behavioral Activation/Inhibition Scale (BAS/BIS; Carver and White, 1994) and the Marlowe-Crowne Social Desirability Scale (MCSD; Crowne and Marlowe, 1960) to measure whether respondents were responding truthfully or were misrepresenting themselves in order to manage their self-presentation. These questionnaires were used to gain basic information about the participants and assess potential differences between the two groups.

### Experimental Task and Mindfulness Session

#### Experiment Time-Line

Subjects had to perform the experiment two times on different days, taking approximately 2.5 h for each. The questionnaires were collected only on the first day of the experiment for each individual. For the task, participants first performed 240-trials of the RSVP task as the pre-test. This was followed by either meditation or rest (see Figure 1) and then a further 240 trials of the RSVP task (post-test). The order in which the experimental sessions employed meditation and rest was balanced across groups and participants.

#### Meditation Session

The instructions for the meditation session were for participants to concentrate on a physical point inside the center of chest (to the right of the heart) and keep the body and mind relaxed. This is one common type of meditation training for the meditator group. If they could not focus on the heart, they were advised to focus on their heartbeat without counting. They were also instructed to try to sense the temperature of the chest and, while trying to focus on the heart, maintain continuous concentration without stress. If there were wandering thoughts, they were suggested to let go of these and shift their attention back to the heart. They were required to keep their eyes closed and sit on a mat with a crossed leg posture during meditation, but they could change to another comfortable posture if they needed to. The meditation instructions were relatively simple, such that any differences between the two groups in carrying these out may relate to factors such as familiarity of finding and concentrating on the focus point.

#### Rest Session

The instructions for the rest condition were to keep body and mind relaxed and just to take a rest without falling asleep. They were allowed to pursue any wandering thoughts or flow of consciousness. As the primary objective was just to take a rest they were asked not to try too hard to think of anything. All subjects kept their eyes closed during the rest condition and sat with a crossed leg posture the same as for the meditation condition.

### RSVP Task

To perform the RSVP task, participants sat on a chair in front of a computer keyboard and screen and were instructed to focus on the central stream of the task (described in more detail below) and ignore the peripheral streams, with the aim of the task being to recognize the red letter in the central stream.

The task (see Figure 2) was adapted from (Chang et al., 2013, 2016; Tsai et al., 2017) and involved presentation of three letter streams on a white background. These were shown on a 23″ LCD monitor with a vertical refresh rate of 120 Hz. There were 24 uppercase letters in each letter stream with letters selected randomly and without replacement. In most cases the letters “H” and “E” were omitted (in cases where they would have been non-targets). When “E” was the target, “H” was omitted, with the second omitted letter selected at random. Similarly, when “H” was the target “E” and a random letter were omitted. Each letter was 1° × 1.3° in size and appeared for 50 ms followed by a 16.7 ms blank interval. Hence a new letter was presented every 66.7 ms (50 + 16.7 ms). In the middle stream, only one red (Commission International de l’Eclairage, *x* = 0.60, *y* = 0.34) target letter was presented, with other letter colors randomly selected, including green (CIE *x* = 0.28, *y* = 0.58), purple (*x* = 0.25, *y* = 0.14), blue (*x* = 0.17, *y* = 0.13) and yellow (*x* = 0.38, *y* = 0.46). Colors were all isoluminant (22 cd/m^2^). The red target appeared randomly among the 15th to 20th letters presented. The peripheral streams were located 3° to left and right side of the middle target stream and all letters were gray except the distractors. One-third of the trials were the TC distractor condition. In these trials, four consecutive peripheral distractor letters (either all in the left stream or all in the right stream) were red—the TC (see Figure 2). Another third of trials were the non-target colored distractor (NTC) condition. In these trials four consecutive peripheral distractor letters (either all in the left stream or all in the right stream) were green. The remaining one-third of trials made up the distractor absent condition, where all letters in the distractor streams were gray.

When performing the task, participants were asked to focus on the central stream of letters and to ignore the peripheral streams with the aim of recognizing the red letter in this central stream. Only 20 letters could be the target (A to J and Q to Z, with other letter restrictions as mentioned previously) and the trial number at which a certain letter was the target was consistent. They had to press a key with the right-hand index finger when the target was one of the letter A to J (i.e., one of the first ten letters of the alphabet), and press another key with their right-hand middle finger when the target was a letter from Q to Z (i.e., one of the last ten letters of the alphabet). Each letter was a target equally frequently. The accuracy of responses was emphasized with it not being necessary to make responses in a speeded manner. Participants performed the 240-trials of the task with a short rest break after completing 120 trials. Approximately 20–25 min were needed to complete all trials. The task was presented in a dimly lit, quiet room with electrical shielding to aid with EEG recording.

### EEG Protocol and Analysis

#### EEG Recording Parameters

During performance of the task, participants wore a 36-channel digital EEG cap (Quik-Cap) with Ag/AgCl sintered electrodes placed in accordance with the International 10/20 system (FP1, FP2, F7, F3, Fz, F4, F8, FT7, FC3, FCz, FC4, FT8, T3, C3, Cz, C4, T4, TP7, CP3, CPZ, CP4, TP8, T5, P3, Pz, P4, T6, O1, Oz, O2, VEOU, VEOL, HEOL, HEOR, A1, A2). The left and right mastoids were used as offline references. The impedances of all EEG electrodes were kept below 5 kΩ. A Neuroscan amplifier (Nuamps) and Neuroscan 4.5 software were used for EEG data acquisition, which was initially acquired with the maximum bandwidth of the amplifier (i.e., DC-260 Hz). The EEG signal was recorded continuously with a sampling rate of 1000 Hz.

#### Event-Related Mode Analysis

EEG data were analyzed by application of an improved method, event-related mode (ERM) for measuring event-related potentials (ERPs; Cong et al., 2009; Hsu et al., 2016). Instead of applying a low-pass (e.g., <30 Hz) or band-pass (e.g., 0.5–30 Hz) filter to the EEG data as is conventional for ERP analysis, ERM employed empirical mode decomposition (EMD) or ensemble empirical mode decomposition (EEMD; Wu and Huang, 2009) to obtain waveforms within a frequency range (e.g., 2–16 Hz). This was employed as this approach can significantly improve the signal-to-noise ratio and consequently have a better sensitivity to stimuli than conventional ERP analysis (Hsu et al., 2016). EMD/EEMD is a data-adaptive method to decompose a series of signals into a set of intrinsic mode functions (IMFs) which typify the local properties of the signals in the time and frequency domain. Moreover, the procedure of EMD/EEMD has been indicated as a bank of natural dyadic filters (Flandrin et al., 2004), by which the data can be decomposed to a finite number of modes (i.e., IMFs) from high to low frequency ranges with minimized distortion of waveforms (Huang et al., 2009). Since an IMF, obtained from EMD/EEMD, has properties of symmetry and zero-crossing, the corresponding instantaneous frequency (IF) can reliably be obtained by applying an improved Hilbert transform (Huang et al., 2009). The EMD/EEMD in together with the Hilbert transform gives the Hilbert-Huang Transform (HHT). Similar to conventional ERP procedures, averaging IMFs across trials gives ERMs (Al-Subari et al., 2015b). Based on the instantaneous frequencies (Huang et al., 2009) of IMFs, ERP components can be extracted by summing ERMs (Cong et al., 2009; Williams et al., 2011; Wu et al., 2012), or using an ERM, within a frequency range (Al-Subari et al., 2015a). This ERM-based ERP component retrieval process may provide better resolution of a specified ERP component because before averaging the waveforms obtained from IMFs are less distorted compared data acquired using conventional filtering. It has been found that the EEMD method results in ERMs with extraordinarily high signal-to-noise ratios and can show a large effect size (Williams et al., 2011). It has also been found that fewer trials and fewer participants are required for ERM, as compared to more conventional ERP analysis (Hsu et al., 2016).

EEG epochs were analyzed from 1000 ms prior to and 1000 ms following the distractor onset. After epoching, Independent Component Analysis (ICA) was performed to remove vertical eye blinks and was followed by artifact rejection with a ± 100 μV threshold for every channel.

**Figure 3:**
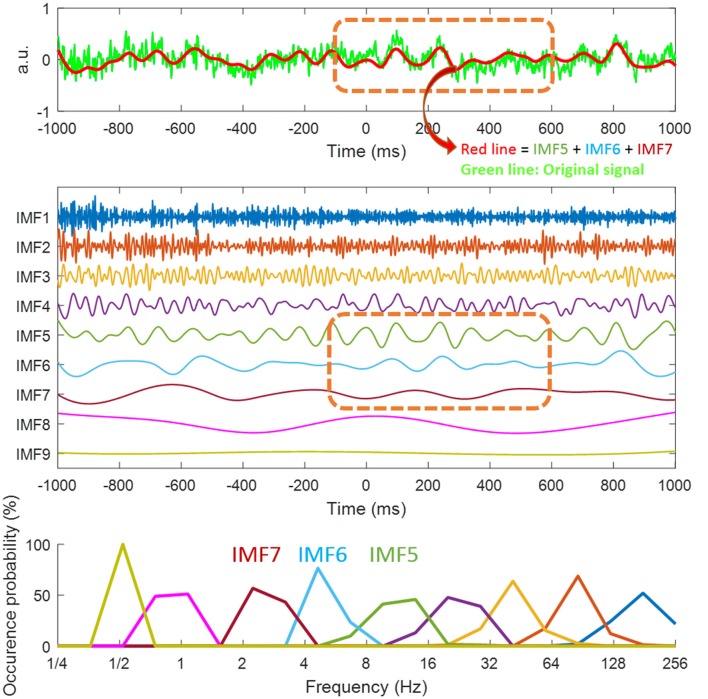
Illustration of intrinsic mode functions (IMFs) from ensemble empirical mode decomposition (EEMD). Decomposition of the signal from the P3/P4 electrodes for target color (TC) conditions (the green line in top panel) through EEMD. We obtained nine IMFs (middle panel). For the first two graphs, the *x*-axis is time with 0 ms being the distractor onset. Different IMFs represented electrophysiological activities in different frequencies. For instance, IMF1 (1st IMF) was high frequency noise, IMF2 and IMF3 represented gamma activity and IMF4 was beta activity. Because the interested event-related potential (ERP) components (i.e., N2pc and Pd) in this study are all within low frequency range, we summed up IMF5, IMF6 and IMF7 (i.e., alpha, theta and delta activity) for the current event-related mode (ERM) analysis (the red line in the top graph). The last IMF (IMF 9) is the trend of the analyzed data. The orange dotted line (graphs 1 and 2) is the time window for ERM analysis. Bottom panel showed the distribution of instantaneous frequencies of each IMF.

After artifact rejection, EEG data of all trials was normalized in each channel by dividing it by its standard deviation, resulting in a unit-free measure of the amplitude. All epoched data were analyzed by EEMD with ensemble size 100 and noise level 0.35 (the ratio of the standard deviation of the adding noise and the standard deviation of the original signal). After EEMD, nine IMFs were obtained. In event-mode analysis, the 5th, 6th and 7th IMFs, which correspond to alpha, theta and delta activity (see Figure 3) were selected and summated as the data for analysis. These IMFs were selected as they represent the activity for the alpha to delta frequency ranges. IMF 4 (see Figure 3) corresponded to beta activity, and the order of the period is around 50 ms. In event-related EEG, components higher than 20 Hz are usually not “phase-locked” across trials so IMF 5 was selected as the upper boundary. For the lower frequency boundary, if the frequency of the IMF is lower than 2 Hz, then period is above 1000 ms, so potentially longer than the time window analyzed in this task. The low frequency band between alpha to delta is typical of ERP components for analysis, especially theta activity for the N2pc component (Dowdall et al., 2012; Al-Subari et al., 2015b; Sawaki et al., 2015). The epochs were averaged according to the distractor types (absent, NTC, TC) and interventions (pre-test, post-meditation and post-rest). We applied the HHT with customized MATLAB (Math Works, Natick, MA, United States) scripts with EEMD program codes from the Research Center for Adaptive Data Analysis of National Central University, Taiwan. SPM8 for MEG/EEG (Wellcome Department of Cognitive Neurology, London, UK) was used for further data processing and statistical analysis.

The N2pc and Pd were calculated through subtracting the mean amplitudes of the ipsilateral electrode from the contralateral electrode with reference to the location of the distractor (NTC and TC). The time window for the N2pc was between 150–250 ms and for the Pd was between 280–380 ms following distractor onset. The statistical analysis of ERM data was consistent with the analysis of the behavioral data. To test for any effects of meditation, a mixed-design analysis of variance (ANOVA) [2 (group (meditator vs. control)) × 3 (intervention (pre-test, post-meditation and post-rest)) × 2 (distractor type (NTC, TC))] was performed.

## Results

### Questionnaire Scores

#### Comparing Meditator to Control Personality Traits

Independent *t*-tests were conducted to evaluate the self-report measures for each questionnaire including STAI-state, STAI-trait, BIS, BAS, MAAS and MCSD (for statistical details, see Table 1). For anxiety levels, the *t*-test for STAI-trait was significant, with the anxiety level in the meditator group lower than in the control group. However, there was no significant difference in state anxiety level between the two groups. The mindfulness trait, reflected by MAAS scores, showed no significant difference between the two groups. The result was contrary to our expectations that meditators might be more mindful than non-meditators. The *t*-test for BIS was significant. The behavioral inhibition system scores in meditators were lower the control group. However, there was no significant difference for BAS between two the groups. For the MCSD, used to measure social desirability, there was no significant difference between the two groups.

**Table 1:** Independent *t*-tests between meditator and control groups on different self-report measures.

Questionnaire	Meditators: mean (std.)	Controls: mean (std.)	*t* value	*p* value
State-trait anxiety inventory: state anxiety	32.92 (8.04)	37.08 (10.49)	−1.60	0.115
State-trait anxiety inventory: trait anxiety	41.15 (7.48)	46.08 (7.64)	−2.35	*0.023
Mindful attention awareness scale	63.12 (9.46)	59.19 (9.75)	1.47	0.147
Behavioral activation scale	39.04 (5.06)	39.69 (4.10)	−0.51	0.611
Behavioral inhibition scale	18.87 (3.61)	21.42 (2.91)	−2.87	*0.006
Marlowe-crowne social desirability scale	16.50 (6.22)	14.31 (4.73)	1.43	0.159

The asterisk (*) indicates statistical significance (p < 0.05).

Based on meditators’ self-report of their amounts of training, years of meditation training was significantly correlated with MAAS scores (*r* = 0.439, *p* = 0.025) with more years of training resulting in higher scores for the mindfulness measure. The time spent in each practice was also correlated to MAAS scores (*r* = 0.442, *p* = 0.026), with longer time spent in each meditation related to more mindfulness. Years of meditation training was also significantly correlated to the time in each practice (*r* = 0.689, *p* < 0.001). Years of practice were not correlated with other self-report measures.

### Behavioral Performance

A mixed three-way ANOVA was conducted to evaluate the hypothesis that meditation state might affect attentional capture, in comparison to the resting state. Additionally, whether the meditator group might have lesser interference by target-colored distractors than control group could be assessed. The three factors of the ANOVA were group (meditator and control), intervention (pre-test, post-meditation and post-rest) and types of distractor (distractor absent, non-target colored distractor and target colored distractor). The factor, intervention, allowed for assessment of any state effects of meditation. Of primary interest was comparison of post-meditation and post-rest performance. Both pre-meditation and pre-rest are the baseline without intervention and they theoretically do not differ. The paired *t*-tests comparing pre-meditation and pre-rest of Absent (*t*_(51)_ = 1.58, *p* = 0.120), NTC (*t*_(51)_ = 1.21, *p* = 0.232) and TC (*t*_(51)_ = −0.651, *p* = 0.518) trials were not significant. Since there was no statistical difference on pre-meditation and pre-rest before interventions, indicating no difference in the beginning of the two different experimental days and both were baselines, these were averaged as “pre-test”.

**Figure 4:**
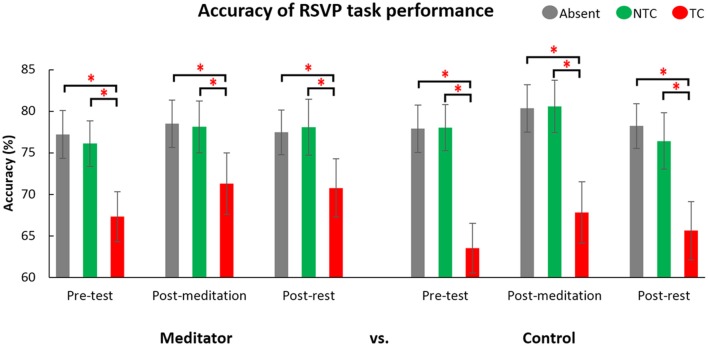
Behavioral performance on the RSVP task for different distractor types and interventions. The gray bars are the distractor absent condition (Absent (grey)), the green bars are the non-target color distractor condition (NTC (green)), and the red bars are target-colored distractor condition (TC (red)). The accuracy of the TC distractor condition was significantly lower than Absent and NTC across groups and interventions of meditation and rest. Error bar indicate 95% confidence intervals. The asterisks (*) indicate statistically significant differences (*p* < 0.05).

**Figure 5:**
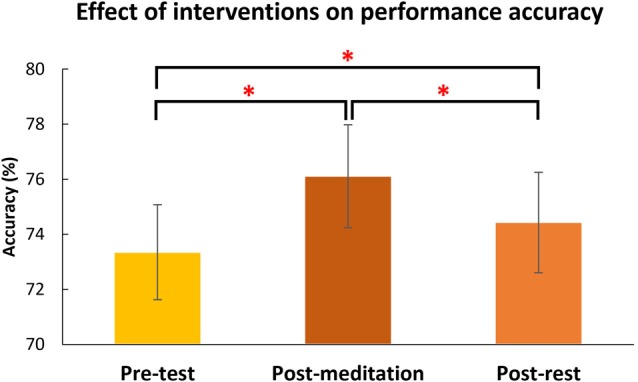
The effect of the interventions on accuracy of the RSVP task. All subjects (across groups and distractor types) performed the RSVP task better after meditation than rest and compared to pre-test. Error bars indicate 95% confidence intervals. The asterisks (*) indicate statistically significant differences (*p* < 0.05).

As expected, accuracy in the TC condition was significantly lower than in the NTC (*t*_(51)_ = 0.629, *p* < 0.001) and distractor absent (*t*_(51)_ = 10.789, *p* < 0.001; see Figure 4) conditions. There was no difference between NTC and distractor absent conditions (*t*_(51)_ = 0.310, *p* = 0.758). These patterns of behavioral results are consistent with previous findings (e.g., Chang et al., 2013, 2016). There was a main effect of intervention (*F*_(1.545,77.243)_ = 9.462, *p* = 0.001, see Figure 5). Paired comparison using LSD showed accuracy post-meditation (*M* = 0.761) was better than post-rest (*M* = 0.744, *p* = 0.038). It showed a larger state effect of meditation than rest. The accuracy post-meditation was better than pre-test (*M* = 0.733, *p* < 0.001), and the accuracy post-rest was better than pre-test (*p* = 0.035). There was a two-way interaction between group and types of distractor (*F*_(1.531,76.566)_ = 6.978, *p* = 0.004). There was no three-way interaction, *F*_(3.064,153.199)_ = 0.63 (Greenhouse-Geisser corrected), *p* = 0.599, and no other significant interactions or differences were found (all *p* > 0.05, for full results of ANOVA, please see Table 2).

**Table 2:** Results of analysis of variance (ANOVA) of accuracy data.

Three-way ANOVA: accuracy	*df*	*F*	*p*	Power
Group (meditator and control)	1	0.19	0.668	0.07
Intervention (baseline, med and rest)	1.545	9.46	*0.001	0.95
Type (Absent, NTC and TC)	1.531	112.93	*<0.001	1.00
Intervention * group	1.545	1.69	0.197	0.30
Type * group	1.531	6.98	*0.004	0.86
Intervention * type	3.064	1.54	0.207	0.40
Intervention * type * group	3.064	0.63	0.599	0.18

The asterisk (*) indicates statistical significance (p < 0.05).

**Figure 6:**
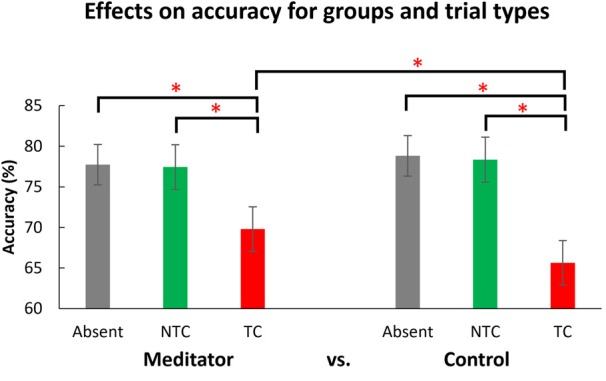
The effects of the different distractor types for the two groups. The accuracy in the target-colored distractor condition (TC) was significantly lower than in the distractor absent (Absent) and non-target color distractor (NTC) conditions. The control groups’ performance in the TC condition was significantly lower than that of the meditator group. The data is the combined data from the two experimental days. Error bars indicate 95% confidence intervals. The asterisks (*) indicate statistically significant differences (*p* < 0.05).

*Post hoc* analysis of the two-way interaction between group and type (see Figure 6) showed meditators’ accuracy for the TC condition was significantly higher than the control group (*t*_(50)_ = 2.10, *p* = 0.041), but no group difference was seen for distractor absent trials (*t*_(50)_ = −0.549, *p* = 0.586) or the NTC condition (*t*_(50)_ = −0.598, *p* = 0.553). These results indicate meditators attentional capture by the TC distractor was less than the control group. In other words, meditators showed less interference by a salient distractor.

### Event-Related Mode (ERM) Analysis

**Figure 7:**
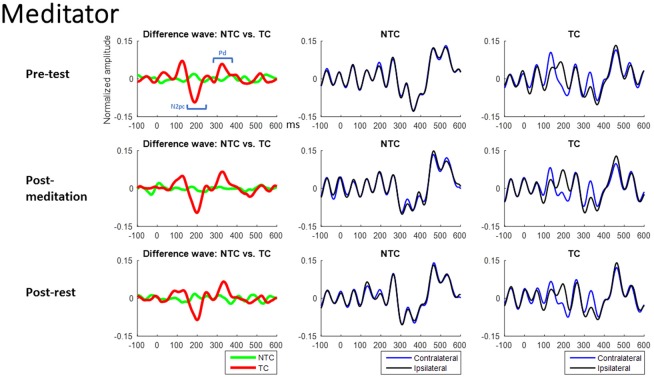
Meditator group’s ERM waveforms from the RSVP task. The Waveforms of left Column were derived from the differences between contralateral and ipsilateral waveforms. The middle Column is the contralateral and ipsilateral waveforms of NTC condition. The right Column is the contralateral and ipsilateral waveforms of TC condition. The *X*-axis is time (ms), and 0 ms is distractor onset. The Y-axis is normalized amplitude. EEG data of all trials in each channel were normalized by dividing it into its standard deviation, therefore resulting in a unit-free measure of the amplitude.

**Figure 8:**
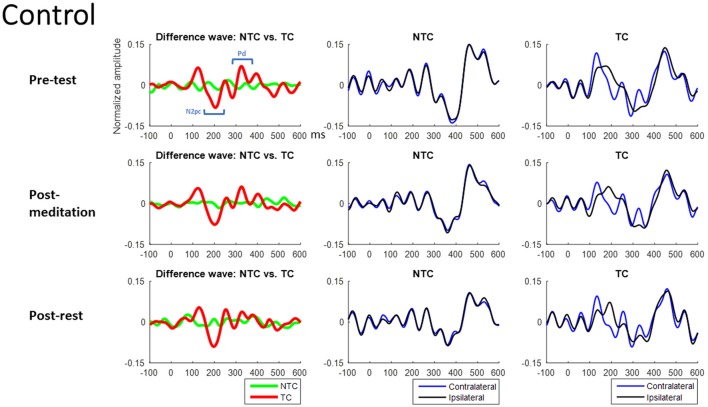
Control group’s ERM waveforms from the RSVP task. The Waveforms of left Column were derived from the differences between contralateral and ipsilateral waveforms. The middle Column is the contralateral and ipsilateral waveforms of NTC condition. The right Column is the contralateral and ipsilateral waveforms of TC condition. The *X*-axis is time (ms), and 0 ms is distractor onset. The Y-axis is normalized amplitude. EEG data of all trials in each channel were normalized by dividing it into its standard deviation, therefore resulting in a unit-free measure of the amplitude.

Figures 7, 8 show the ERM waveforms from the RSVP task. Analysis was of the same structure as the accuracy analysis carried out on the behavioral data, using a mixed three-way ANOVA. The three independent variables in the statistical analysis were: (1) group of subjects (mediators or non-meditators); (2) intervention (pre-test, post-meditation and post-rest); and (3) distractor type (NTC and TC) in the RSVP task.

#### ERM: N2pc (150–250 ms, see Figure 9)

There was a main effect of distractor type (*F*_(1,50)_ = 65.71, *p* < 0.001). The *post hoc* test showed the N2pc for TC trials was significant smaller than NTC trials (*p* < 0.05). There were no other interactions or main effects (all *p* > 0.05, for full results of the ANOVA, please see Table 3). This suggests contingent reorienting happened in the TC condition, with participants shifting attention to the TC distractor but the attentional deployments to salient distractors did not differ significantly for the two groups. The significant effects of intervention indicated the amplitude of N2pc was smaller during the experimental day of meditation. However, we did not observe an interaction between sessions and interventions.

**Figure 9:**
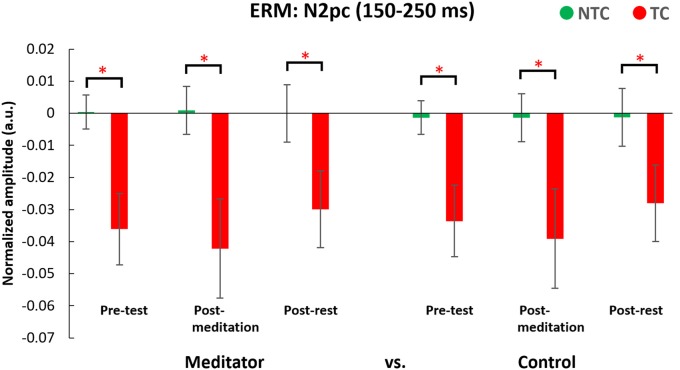
Result for the N2pc component ERM analyses. N2pc values were significantly smaller in the TC condition than the NTC condition. Waveforms were derived from the differences between contralateral and ipsilateral waveforms. Error bars indicate 95% confidence intervals. The asterisks (*) indicate statistically significance differences (*p* < 0.05).

**Table 3:** Results of ANOVA of event-related mode (ERM; N2pc) data.

Three-way ANOVA: ERM (N2pc)	*df*	*F*	*p*	Power
Group (meditator and control)	1	0.01	0.935	0.05
Intervention (baseline, med and rest)	2	2.22	0.113	0.44
Type (NTC and TC)	1	65.71	*<0.001	1.00
Intervention * group	2	<0.00	1.000	0.05
Type * group	1	0.25	0.620	0.08
Intervention * type	2	2.93	0.058	0.56
Intervention * type * group	2	0.02	0.976	0.05

The asterisk (*) indicates statistical significance (p < 0.05).

#### ERM: Pd (280–380 ms, see Figure 10)

The three-way ANOVA of the Pd data showed a main effect of distractor type (*F*_(1,50)_ = 42.27, *p* < 0.001) indicating Pd amplitude in the TC condition was larger than for the NTC and there was a significant two-way interaction (*F*_(1,50)_ = 5.83, *p* = 0.019), indicating the neural patterns for responding to the target-colored distractor were significantly different between the two groups (see Figure 11). In *post hoc* analysis of the two-way interaction between groups and types, the meditators’ Pd for the TC condition was significantly larger than the control group (*t*_(50)_ = 2.193, *p* = 0.033), but no difference were seen in the NTC condition between the two groups (*t*_(50)_ = −0.110, *p* = 0.912). The larger Pd of the meditator group suggests attentional suppression to TC distractors significantly differed from that of the control group. There were no other statistically significant interactions or main effects (*p* > 0.05, for full results from the ANOVA, please see Table 4).

**Figure 10:**
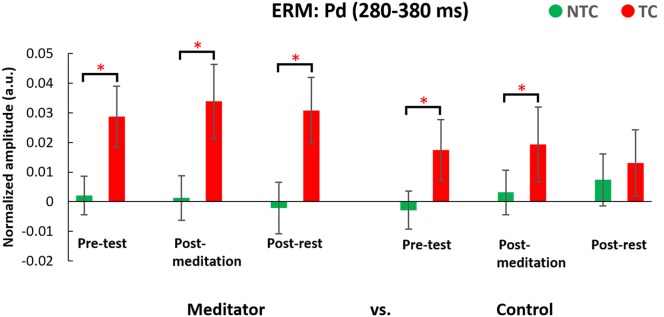
Result of the Pd component ERM analyses. Waveforms were derived from the differences between contralateral and ipsilateral waveforms. Error bars indicate 95% confidence intervals. The asterisks (*) indicate statistically significance differences (*p* < 0.05).

**Figure 11:**
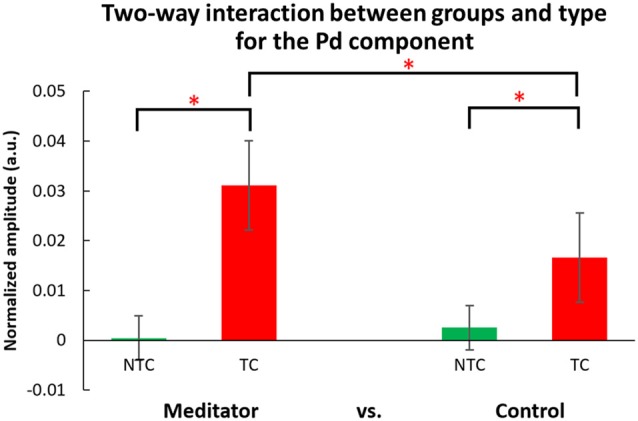
Two-way interaction between group and type for the Pd component. The Pd component (280–380 ms) for the target-colored distractor condition (TC) was significantly larger than for the non-target color distractor (NTC) condition. The meditator group’s Pd in the TC condition was significantly larger than the control group. However, the Pd of NTC did not differ significantly between groups. In this figure, only the two-way interaction of groups and distractor types is shown, and the factor of interventions (pre-test, post-meditation and post-rest) was combined. Error bars indicate 95% confidence intervals. The asterisks (*) indicate statistically significance differences (*p* < 0.05).

**Table 4:** Results of ANOVA of ERM (Pd) data.

Three-way ANOVA: ERM (Pd)	*df*	*F*	*p*	Power
Group (meditator and control)	1	2.97	0.091	0.39
Intervention (baseline, med and rest)	2	0.64	0.531	0.15
Type (NTC and TC)	1	42.27	*<0.001	1.00
Intervention * group	2	0.27	0.767	0.09
Type * group	1	5.83	*0.019	0.66
Intervention * type	2	0.41	0.666	0.11
Intervention * type * group	2	1.55	0.218	0.32

The asterisk (*) indicates statistical significance (p < 0.05).

## Discussion

The present study investigated differences between meditators and non-meditators, as well as state effects of meditation, on attentional control and contingent reorienting by use of different distractors in an RSVP paradigm in conjunction with electrophysiological recording. It was predicted that meditators would show better performance under conditions with potential distractor interference because of better attentional suppression in this group compared to the control group. A major finding of the current study was that both a group difference and an effect of meditation induction could be observed for the attentional capture task. Immediately after completing meditation there was increased accuracy in identification of targets for both groups and for all distractor types, compared to both baseline and the rest induction condition. This was irrespective of the group participants belonged to. The group differences across interventions suggests that efficiency of attentional suppression of the target-like distractor for the meditator group was significantly better than the control group. Meditators’ Pd of ERM during the TC condition was larger than the control group, and this is consistent with meditators having better attentional suppression when the target-like distractors were presented.

### Meditation Effects on the RSVP Task

The present findings are consistent with previous evidence regarding the effects of distractors on RSVP performance (Chang et al., 2013, 2016; Tsai et al., 2017), with accuracy significantly lower in the TC distractor condition than the NTC and distractor absent conditions. This indicates that only the lateral TC distractors, which induced contingent reorienting, interfered with subjects’ ability to recognize the central target. The NTC distractors, which resulted in no significant difference in performance compared to the distractor absent condition, did not induce contingent reorienting. We also observed that overall accuracy both post-meditation and post-rest were higher than the pre-test condition, which could have been a practice effect, a beneficial effect of both interventions (to some degree), or a combination of the two.

The main questions we asked in this study were: (1) whether meditators were different from non-meditators in the measures obtained; and (2) whether the short interventions of meditation affected performance compared to rest. The behavioral data showed a two-way interaction between group and distractor types. *Post hoc* tests indicated meditators’ performance was better when target-colored distractors were presented than for the same type of trials for the control group irrespective of the intervention. Second, we observed a state effect of meditation, with differences between the effects of meditation and rest. Overall task accuracy post-meditation was significantly higher than both post-rest and pre-test, with post-rest also better than pre-test. However, the state effect of short meditation intervention was not specifically beneficial to certain distractor conditions or to one group and not the other. In other words, the meditation intervention had no effect on participants’ abilities to control attentional capture among different distractor types (i.e., no interactions between intervention and any other factors). This study therefore found a phenomenon of a state effect of meditation, similar to recent studies involving people without meditation experience. For example, in such participants trained after brief instructions, and immediately performing attentional or emotional tasks, significant state effects of meditation were seen (e.g., Colzato et al., 2015; Fennell et al., 2016; Lin et al., 2016).

This study also attempted to identify the neural mechanisms underlying any effects of meditation by use of electrophysiological recording. Event-mode analysis of data from the parietal area, along the lines of previous studies (Chang et al., 2016; Tsai et al., 2017), showed a significant N2pc component, usually indicating attentional deployment, to target-colored distractors (Luck and Hillyard, 1994a,b; Sawaki and Luck, 2014), but no N2pc component in the NTC distractor condition. However, there was no significant difference in N2pc amplitude between the two groups, or between the meditation and rest interventions. We did find the Pd amplitude, which is an index of attentional suppression (Hickey et al., 2009; Sawaki et al., 2012; Sawaki and Luck, 2014), was different between the two groups. Results showed meditators’ Pd amplitudes were larger when there was a target-colored distractor, compared to the control group across interventions. This might suggest that higher accuracy in the TC distractor condition for the meditator group was due to better attentional suppression rather than different attentional deployment to the TC distractor. However, ERM results did not reveal a state effect of meditation vs. rest, with no difference between post-meditation and post-rest for the Pd component.

The experimental design employing meditation and rest for 30 min was employed to observing the immediate effects of these interventions, with the expectation that post-meditation performance would be better than pre-test performance. In line with these expectations, there was a main effect of intervention (overall accuracy, combining three distractor types: post-meditation > post-rest > pre-test). In short, meditation for 30 min was associated with significantly better performance than rest for 30 min for both groups. However, this effect did not interact with other factors (i.e., group or distractor types). This suggests the state effects of meditation and rest were the same for both meditators and non-meditators. The specific question we asked is whether meditation intervention alters the control of attentional capture. The results showed that a single session of meditation had no significant effect on attentional control. The performance on the attention task was better following a single session of meditation, and this effect seemed unrelated to the control of attentional capture, which would have been indicated by an interaction between intervention and distractor type. According to Zanesco et al. (2016), meditators engage in less mind wandering following meditation training, and intensive meditation training reduces mind wandering among meditators when they need to maintain attention during a complex cognitive task. Additionally, Brandmeyer and Delorme (2016) showed meditation practice decreased the susceptibility of the mind to wandering and maintained both internal and external orientation of attention. Following just a simple introduction to meditation, participants might tend to have fewer wandering thoughts due to a period of concentrating on the object of the meditation. However, this effect improved overall attention rather than producing an effect for specific distractor conditions.

### Limitations of the Study

There are a number of factors that should be remembered when considering the results found here. Many factors may interact with the effects of meditation, such as personality traits, the identity of the meditation trainer, and group dynamics during training (Tang and Posner, 2013; Lutz et al., 2015; Tang et al., 2015). To address this, at least partially, all participants were required to complete several questionnaires measuring mindfulness levels, anxiety, motivation (behavioral activation and inhibition), and social conformity which might interact with meditation and neurobiological mechanisms (Vago and Silbersweig, 2012). The participants were relative homogeneous, which meant that factors such as occupation, marriage and long-term life experiences were less likely to affect the results. Interestingly, the control group had a mindfulness level that wasn’t significantly different from the meditator group which means the behavioral and neurophysiological data might have affected by meditation skill through training or lower anxiety levels, rather than personality-related mindfulness levels. Because the two groups were different in anxiety level, an alternative to the interpretation that improvement in attentional suppression to TC distractors for the meditator group was due to meditation skill based on training could be that it was due to lower anxiety levels. In the future, a longitudinal study might assess this interpretation more precisely.

There were also some variables that could not be controlled in the study, such as the quality and quantity of meditation training of the meditation group (Vago and Silbersweig, 2012; Tang and Posner, 2013). According to Tang and Posner (2013), for instance, it might be that meditators employed distinct efforts in different stages of meditation. Additionally, the environment was not a familiar place for them to meditate. This study averaged all meditators’ data from different stages of meditation when analyzing effects. However, it was extremely difficult to measure and define individuals’ subjective feelings and quality of meditation. In future, addressing these limitations would be beneficial.

As this was a cross-sectional study, we could not preclude the possibility that there might be pre-existing differences in meditators’ brain, their interest in meditation, or their personality or temperament independent of any effects of meditation training (Tang and Posner, 2013; Tang et al., 2015). Since we wanted to assess meditation effects based on long-term training and practice from meditators, it was difficult to have an active control, such as 3-year relaxation training or reading group participation. Nevertheless, the experimental design in this study executed interventions of meditation and rest 30 min on different experimental days even for the control group who had no meditation experience. Hence, we still had an opportunity to observe the state effects of meditation and whether this produced any effect on behavioral performance or brain electrophysiological measures.

## Conclusion

This study has demonstrated first, that meditators show better attentional control under a condition with a target-like distractor and second, that there was a general state effect of meditation with a short meditation period improving attentional performance relative to rest for both groups. Meditators had better behavioral performance in the target-like distractor condition and better attentional suppression than the control group. However, more investigation is needed to examine whether meditation training might be an appropriate intervention for people who have problems with attentional control. Future research should investigate the meditation stage and assess its neural dynamics to better inform how meditation modulates behavior and physiology in detail.

## Author Contributions

S-YT designed the study, collected and analyzed the data, and was involved in preparation of the manuscript. SJ was involved in the study design, preparation of the manuscript, data interpretation and revision of the manuscript. C-FC programmed the experimental stimuli and codes for data analysis. NM was involved in editing and revision of the manuscript. W-KL was involved in data analysis and revision of the manuscript. C-HJ was involved in the study design, data interpretation and revision of the manuscript. All authors have approved the final version of the manuscript and are accountable for the work described.

## Conflict of Interest Statement

The authors declare that the research was conducted in the absence of any commercial or financial relationships that could be construed as a potential conflict of interest.
